# Limited catching bias in a wild population of birds with near-complete census information

**DOI:** 10.1002/ece3.1623

**Published:** 2015-07-29

**Authors:** Mirre J P Simons, Isabel Winney, Shinichi Nakagawa, Terry Burke, Julia Schroeder

**Affiliations:** 1 Department of Animal and Plant Sciences, University of SheffieldSheffield, S10 2TN, UK; 2 Evolution & Ecology Research Centre, School of Biological, Earth and Environmental Sciences, University of New South WalesSydney, NSW, 2052, Australia; 3 Department of Zoology, University of OtagoPO Box 56, Dunedin, 9054, New Zealand; 4 Evolutionary Biology, Max Planck Institute for OrnithologySeewiesen, DE-82319, Germany

**Keywords:** Capture methods, house sparrow, island populations, methodology, ornithology, trappability

## Abstract

Animal research often relies on catching wild animals; however, individuals may have different trappability, and this can generate bias. We studied bias in mist netting, the main method for catching wild birds. The unusually high resighting rate in our study population—house sparrows (*Passer domesticus*) on Lundy Island (England)—allowed us to obtain accurate estimates of the population size. This unique situation enabled us to test for catching bias in mist netting using deviations from the expected Poisson distribution. There was no evidence that a fraction of the birds in the population consistently remained uncaught. However, we detected a different bias: More birds than expected were captured only once within a year. This bias probably resulted from a mixture of fieldworkers sometimes ignoring rapid recaptures and birds becoming net shy after their first capture. We had sufficient statistical power with the available data to detect a substantial uncaught fraction. Therefore, our data are probably unbiased toward catching specific individuals from our population. Our analyses demonstrate that intensively monitored natural insular populations, in which population size can be estimated precisely, provide the potential to address important unanswered questions without concerns about a fraction of the population remaining uncaught. Our approach can help researchers to test for catching bias in closely monitored wild populations for which reliable estimates of population size and dispersal are available.

## Introduction

Animal research in general, and specifically ornithological research, often relies on catching individuals from a group for monitoring and/or experimental purposes. In particular in the wild, but also in captivity, this procedure can generate so-called catching bias. For example, population sizes can be underestimated, or experiments can be biased by pseudoreplication or by only assessing a subset of a captive or wild population (Chao 1987; Milinski 1997; Biro 2013; Winney et al. 2015). Individuals may vary in the ease with which they are caught (Biro and Dingemanse 2009), and this trait may causally, or coincidentally, covary with other, biologically relevant traits. Indeed, in captive and wild populations, capture order and phenotypic traits have been found to be positively correlated with, for example, immune functioning (Birkhead et al. 1998), sexual signal expression (Birkhead et al. 1998; Moreno-Rueda 2003), age (Moreno-Rueda 2003), and growth rate (Biro 2013), suggesting that individuals of higher quality have a lower propensity to be caught. Such heterogeneity in catching propensity can severely bias the results and conclusions. For example, less explorative great tits (*Parus major*) avoid nestboxes that have a video camera fitted, thus biasing recordings toward bolder individuals (Stuber et al. 2013). Such biases will be exceptionally misleading when examining the fitness correlates of behaviors, such as animal personality (Biro and Dingemanse 2009; Stuber et al. 2013). When, for example, the shiest individuals remain unseen, this limits accurate estimation of the fitness landscapes of personality traits.

Different methods of catching or observing animals are predicted to have differential levels of catching/observation bias, and this has been a continuing topic of study in ornithology (Pienkowski and Dick 1976; Hansrote and Hansrote 1991; Bauchau and van Noordwijk 1995; Domenech and Senar 1997). Here, we investigate an ubiquitously used tool in ornithology, mist netting of small birds (Karr 1981; Jenni et al. 1996; Lövei et al. 2001). Passerines are often used as model species, and data resulting from catching passerines form the basis of a large part of our knowledge on the ecology and evolution of wild vertebrates. Mist netting is often the preferred capture method (Karr 1981; Peach et al. 1996), because it is efficient and has been shown to impose a very low risk of injury to the birds (Spotswood et al. 2012). However, we do not know whether personalities or other effects bias catching success when using mist nets. Even without considering net detection and evasion, the ability to escape the net once an individual bird hits a net—which can be as high as 37% (Lövei et al. 2001)—could vary among individuals.

Considering the importance of data from captures of birds in the study of ecology and evolution, the assessment of any potential bias in data from natural populations is crucial. However, to our best knowledge, not many studies on this topic in wild populations exist (see Stuber et al. 2013). Models to estimate population size from recaptures of marked individuals have included heterogeneity among individuals in the propensity to be caught and covariates associated with this propensity and temporal effects due to trap shyness (Chao 1987; Huggins 1989; Chao et al. 1992; Roche et al. 2013). Such statistical approaches increase the precision of population size estimates, a key parameter in ecology, and can examine predictors of catching propensity, but are not necessarily an informative and critical test of catching bias. Part of the heterogeneity in the estimated catching propensity will also arise from variation in dispersal patterns of marked individuals, and immigration and emigration of marked and unmarked individuals to and from the population.

These uncertainties limit estimation of catching bias in the wild in most study populations. Individual catching propensity can be reliably estimated in captive populations, because uncaught individuals will be known or eventually caught. Studies in captivity have revealed significant repeatability of capture order in Zebra Finches (*Taeniopygia guttata*) (Birkhead et al. 1998) and Mountain (*Poecile gambeli*) and Black-capped Chickadees (*Poecile atricapillus*) (Guillette et al. 2010), and capture order is correlated to individual capture time in captive House Finches (*Haemorhous mexicanus*) (Mateos-Gonzalez et al. 2014). These individual consistencies in behavior do suggest that catching bias in the wild could be common. Here, we use data from a natural population that does not suffer from the common drawbacks of studies on wild populations to estimate catching bias in passerines, namely unknown population size, a substantial amount of unmarked individuals, and unknown rates of immigration/emigration. The Lundy House Sparrow (*Passer domesticus*) population has been intensively monitored since 2000 (Schroeder et al. 2011), which has resulted in near-perfect yearly resighting rates of 0.96 (M. J. P. Simons et al., unpubl. ms.), estimated using Bayesian Survival Trajectory Analysis (Colchero et al. 2012), and 0.91 in a smaller sample estimated with MARK (Schroeder et al. 2011). We have quantified rates of immigration and emigration, and found them to be negligible (Schroeder et al. 2015), likely due to a combination of the considerable distance from the nearest shoreline of 19 km and relatively poor flying ability of the house sparrow, especially in open and windy terrain. These are exceptionally precise estimates for a wild bird population. More importantly, knowledge on these quantitative parameters gives us the unique opportunity for estimating population size precisely. The Lundy sparrow data thus allow us to thoroughly evaluate and discern potential catching bias using deviations from the expected Poisson distribution of captures. Therefore, this population is exceptionally well suited to test for any catching bias arising from physiological and behavioral differences among individuals that might influence their propensity of being caught and/or from methodological issues, such as oversampling certain areas.

## Methods

### Study population

The study of breeding and survival of house sparrows on Lundy Island (<5 km long, 0.7 km wide; 51.10°N, 4.40°W, UK) has been carried out systematically since 2000 (Cleasby et al. 2011). The closed nature of this population allowed us to capture, measure, sample, and individually mark (using color and metal rings) nearly every adult sparrow on Lundy Island (descriptions of the study can be found in Hsu et al. 2015; Schroeder et al. 2015). Each year, all breeding attempts were closely monitored. This study focuses on catching undertaken during the winter (Nov–Feb) from 2000 to 2011. Annual catching trips focussing specifically on catching sparrows were made for between 5 and 10 days once or twice between November and February every year. During these visits, mist-nests were erected in all locations that the sparrows usually inhabit, primarily in and around the barns and sheds where the nestboxes are located. Lundy is an island of bare rock, and open fields of heather, moss, and lichen. Sparrows are only observed in the small part of the island where there are human habitations, especially around the farm buildings. This is where we focussed our capture efforts. Additional data comes from the Lundy Field Society's general bird surveys, where mist nets are placed in the small wooded area of Lundy (Milcombe valley) with the aim of capturing migratory passerines. The winter captures constitute the main data that we tested for capture bias.

### Population size

To precisely estimate the annual population size, we used the captures in winter and in summer, *ad libitum* live sightings of individual birds using individual color rings, sightings from social parentage assignment of broods using video recordings and color rings as identification (Nakagawa et al. 2007; Schroeder et al. 2013), and whether the bird appeared as a parent in the genetic pedigree (Schroeder et al. 2012; Hsu et al. 2014) from the whole year (defined as from March to next February for each year). We believe that the sum of these observations reliably describes the annual population sizes for two reasons. (1) Dispersal to and from Lundy is generally thought to be rare because Lundy is 19 km from the closest UK shore and house sparrows rarely disperse over the sea (Bengtson et al. 2004; Magnussen and Jensen 2009). Supporting this notion, our genetic data show that between 2000 and 2011, there were only four immigrant birds that had a genotype that was not assignable to parents from the island (Schroeder et al. 2015). (2) We assigned parentage using DNA samples collected from any sparrow on Lundy with unusually high precision (Hsu et al. 2015). Both of these features are only possible if all adults are DNA sampled and genotyped.

### Comparison to expected Poisson distribution

Captures in mist nets per individual per year should follow a Poisson distribution if the chances of being caught are equal among individuals. Thus, if there is a deviation from the Poisson distribution, we can conclude that individuals vary in their trappability. The rate of capture in the population is equal to the variance of the expected Poisson distribution (Ludwig and Reynolds 1988). In our case, the expected Poisson distribution is defined by the total number of captures made in winter divided by the estimated population size. As expected in bird populations, juvenile mortality was substantially higher than adult mortality (M. J. P. Simons et al, unpubl. ms.). Furthermore, the population of juveniles alive at the time of our catching efforts will largely be determined by fluctuations in juvenile mortality, and the number of juveniles produced in the preceding summer. Therefore, we estimated catching bias in adults, excluding juveniles from the mist-net captures. The few rare cases when birds were seen as postfledgling, but not when they were a chick (∼9%), and could therefore not be assigned as juvenile, were assigned as being adult. Subsequently, we corrected the population size of this group of birds (hereafter “adults”) for mortality (see below).

**Figure 1:**
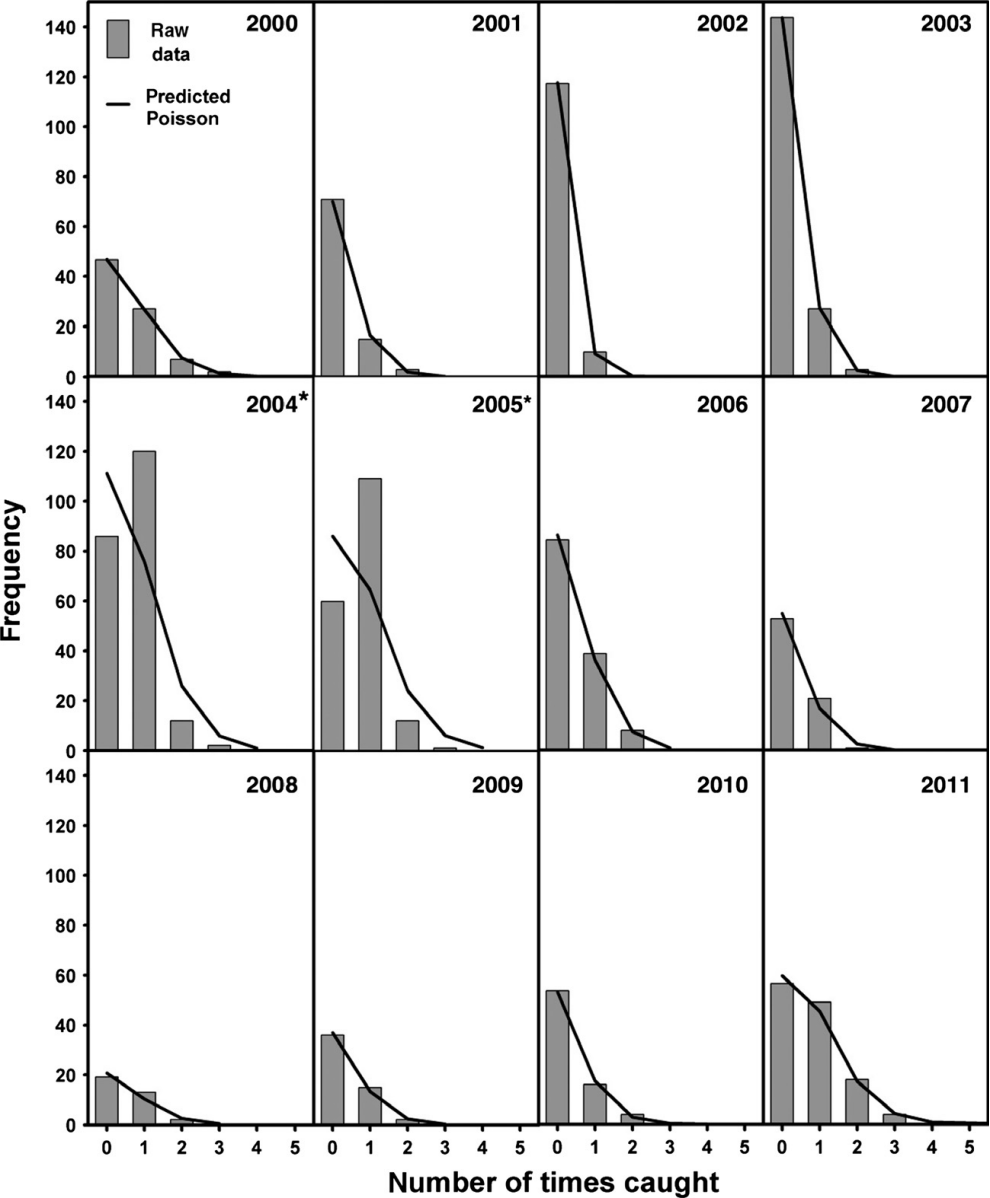
Frequency of the number of times an individual is caught per year compared with the expected Poisson distribution (line). Winter year is the calendar year in which winter starts. Asterisks indicate significant deviations from the expected Poisson distribution.

Across years, most visual sightings were made during the breeding season, while the average winter catching effort was conducted in November (5 months later). We estimated the population at the time of the winter catch to be the population size of adults during the preceding breeding season *minus* the expected adult mortality that occurred during the preceding 5 months (5/12 multiplied by the number of birds not resighted in the subsequent year). This assumes a constant rate of adult mortality. Furthermore, we corrected this estimate for the unseen fraction of the population by multiplying it by the inverse of our best estimate of the adult resighting probability (1/0.96). This is a relatively simplistic estimation of the population size of adults, but is the best estimate available. Note, however, that being able to make even such simplistic estimates of adult mortality is unusual because in most wild populations, a full census, like ours, is unavailable. Lacking census information prevents the accurate estimation of population size and/or mortality to correct for deaths up to a catching event. Using the estimated annual adult population sizes and the actual adult captures using mist netting per winter, we generated the predicted Poisson distribution per year (Table 1, Fig. 1).

**Table 1:** Adult population sizes and the number of captures made each year, used to estimate the predicted Poisson distributions. The number of unique adults that were caught in each year is also depicted and relate to the number of recaptures as depicted in Figure1. Winter year is the calendar year in which winter started

Winter year	Total number of adult captures	Number of unique adults caught	Adult population size
2000	47	36	83
2001	21	18	89
2002	10	10	127
2003	33	30	174
2004	150	134	220
2005	136	122	182
2006	55	47	131
2007	23	22	75
2008	17	15	34
2009	19	17	53
2010	24	20	74
2011	97	71	128

We evaluated the fit of the actual number of captures per individual with the expected Poisson distribution using observed versus expected goodness-of-fit chi-square tests (Ludwig and Reynolds 1988). The last category in these comparisons—the birds caught most often—contained between one and eight individuals. Low expected values (< 3) in this last cell violate the chi-square approximation, but can be pooled in a more conservative test (Ludwig and Reynolds 1988). However, note that we left such cells unpooled in our analyses, and our tests are therefore sensitive rather than conservative. Note that these tests also included the category of adults that were not captured (the population size *minus* the total of unique individuals caught). We evaluated these statistics per year and across all years.

Next, we performed statistical power simulations (written in R, code available upon request) to investigate the uncaught fraction that could be detected with the adult population sizes observed across the years in our study. We modeled the adult population size ranging from 75 to 165 adult individuals, which are the 25% and 75% percentiles of population sizes observed (Table 1). This allowed us to estimate the type II error in our data, that is, the failure to detect true bias. We varied the uncaught fraction, those individuals with a capture rate of zero, of the population in these simulations from 0.05 to 0.70, while holding the mean probability of capture across all years of the study fixed at ∼0.4 by adjusting the rate at which the rest of the population was caught accordingly, thereby assuming a bimodal distribution of catching probability with individuals not caught (a chance of 0) and the other individuals in the population caught at equal rate, adjusting this equal rate such that the average rate was ∼0.4 across the parameters tested. Each parameter set was simulated 10,000 times, and the statistical power calculated as the proportion of these simulations in which a statistically significant (*α =* 0.05) deviation from the expected Poisson was detected.

## Results

In the 12 years of the study (2000–2011), capture rates differed significantly from the expected Poisson distribution only in 2004 and 2005 (*P* < 0.001, Fig. 1). The chi-square test across all years suggested that the observed pattern of captures deviated from the expected Poisson (*χ*^2^_38_ = 96, *P* < 0.001). Note, however, that these deviations from the expected Poisson distributions do not stem from a subpopulation of birds evading capture in winter. Rather, in years 2004 and 2005, there was an overrepresentation of birds that were caught only once or twice, while the Poisson distribution predicted them to be captured more often, resulting in an underrepresentation of birds caught multiple times within a winter.

**Figure 2:**
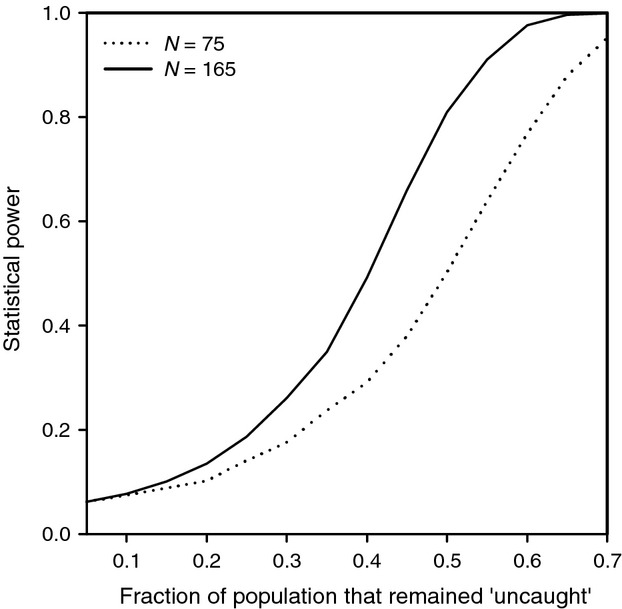
Power simulations of deviations from the expected Poisson with an annual catching rate of ∼0.4 (the mean rate across the years of the study) that could be detected when the fraction of the population that remained uncaught (rate of zero) was varied. Statistical power for the 25% and 75% quantiles (*N* = 75, *N* = 165) of adult population sizes available in this study becomes moderate to high when a fraction of 0.4–0.7 remains uncaught.

Power analyses (Fig. 2) indicated that with the adult population sizes we used (75 and 165), we could detect deviations from a Poisson distribution with reasonable statistical power (> 80%), when we assumed that the uncaught fraction of the population was high (> 0.45).

## Discussion

The closed population, very high percentage of marked individuals, and subsequent high resighting probability of the house sparrows on Lundy permit very accurate estimates of the population size. This allowed for the first assessment of catching bias in a wild passerine without making broad assumptions about population structure and dispersal. Under such conditions in a closed population, or open populations where dispersal is estimated accurately and separately from mortality, deviations from the expected Poisson distribution can be used to estimate catching bias. If such estimates cannot be made reliably, then catching bias could be evaluated using a subset known to remain in a location, for example, through breeding records. More precise estimates of location could also be used through the use of passive integrated transponder (PIT) tagging, now widely available for a growing number of study populations (Schroeder et al. 2011; Aplin et al. 2013), to estimate the size of the population subjected to catching efforts and, subsequently, evaluate catching bias within this subset.

Our analyses indicate significant catching bias in two of the twelve years included in the study. This bias was not because some individuals remained uncaught, however, but because the number of recaptures within a certain year was lower than expected. Such a pattern can result from unrecorded recaptures. Recapture was on occasion not recorded when a bird was caught in rapid succession, that is, set free and then caught again at the next net check, or later that day. Such a gap in consistent note taking would result in the numbers caught repeatedly being smaller in the data set than in reality. Note that the aim of our catching efforts was not specifically to estimate catching bias. Therefore, although our standard protocol is to note the capture of each individual, sometimes birds that were recaptured in relatively close succession may have been released without being recorded.

This bias of on overrepresentation of individuals being only caught once can also be the result of a short-term behavioral response by the birds to a catching event, that is, the bird becomes trap(net)-shy, such that this response reduces the probability of a bird being recaptured multiple times. Such behavior, net shyness, is likely to be common, and its duration will bias subsequent recapture in the short term, as our results indicate, or in the long term such as across an individual's lifetime as shown in cliff swallows (Roche et al. 2013). Such longer term effects are not supported by our results because the fraction that remained uncaught in the population was either on par with the expected Poisson or lower, indicating that at the start of each winter each individual had the same chance of being caught at least once by our mist netting efforts. We therefore tentatively conclude that our results reject the hypothesis that catching bias results in a fraction of the population remaining uncaught in our population during yearly catching efforts and that the underrepresentation of individuals caught is either due to the experimental procedures or due to their biology as outlined above.

Importantly, such a bias in annual recaptures does not affect studies of most behavioral ecological questions. However, if this heterogeneity is not estimated, it may bias estimates of population size (Chao et al. 1992) and population dynamics (Pollock et al. 1990), key parameters in ecology, evolution, conservation, and population ecology. Severe bias might also reduce the precision of estimates in studies focussing on within-individual longitudinal changes in traits (van de Pol and Verhulst 2006).

Our power analyses indicate that while we have statistical power to detect relatively large biases that could strongly affect the interpretation of results, we do not have sufficient power to detect smaller biases in our dataset (Nakagawa and Foster 2004). However, we assume the practical and biological importance of such a small bias to be relatively low, although future extension of our study and/or testing for catching bias resulting in a fraction of uncaught individuals in larger populations could also exclude the possibility of smaller catching biases.

Deviations from the expected Poisson distribution permit the evaluation of bias arising from an uncaught fraction of the population (Biro and Dingemanse 2009; Stuber et al. 2013). Estimates of catching bias obtained from different capture methods can also indicate catching bias. Such comparisons, however, leave the direction of bias (i.e., whether one method is less biased) and the possibility of an unseen fraction in the population unaddressed. For example, when comparing cannon netting with mist netting in waders (Pienkowski and Dick 1976), including Redshanks (*Tringa totanus*) (Insley and Etheridge 1997), and clap netting, a Yunick platform trap and mist netting in serins (*Serinus serinus*) (Domenech and Senar 1997), all comparisons indicated that juveniles were overrepresented in captures from mist netting. However, this does not necessarily mean that mist netting is biased, as it could alternatively be that the reference trapping is biased toward adults. Comparisons between trapping methods simply cannot indicate whether there is a trapping bias in the reference category and/or whether a fraction of the population remains uncaught. Traits that are suspected to covary with trappability can also be tested against catching rate within a trapping method (Huggins 1989). Such models can reveal that certain types of individuals evade capture, for example, in great tits (*Parus major*) (Bauchau and van Noordwijk 1995) and cliff swallows (*Petrochelidon pyrrhonota*) (Roche et al. 2013), older individuals were less likely to be caught using mist netting. Recapture models have been expanded to also estimate heterogeneity in the population from unknown causes (Chao et al. 1992), but this also encompasses heterogeneity originating from other causes such as dispersal or death if a complete current census is not available.

We therefore hope that our method of estimating catching bias using comparisons to a predicted Poisson distribution will prove helpful to others working on populations with reasonably reliable estimates of dispersal. These estimates could provide information on whether catching bias is common and whether a fraction of the population remains uncaught. In addition, such studies will clarify whether catching bias in the wild affects the accuracy and applicability of conclusions drawn from studies of wild populations. This is important because our current knowledge of the extent and nature of catching bias largely stems from theoretical or captive studies (Biro and Dingemanse 2009; Biro 2013), with limited data from the field.
